# Interferons in Pain and Infections: Emerging Roles in Neuro-Immune and Neuro-Glial Interactions

**DOI:** 10.3389/fimmu.2021.783725

**Published:** 2021-11-05

**Authors:** Ping-Heng Tan, Jasmine Ji, Chun-Chang Yeh, Ru-Rong Ji

**Affiliations:** ^1^ Department of Anesthesiology, Chi Mei Medical Center, Tainan City, Taiwan; ^2^ Neuroscience Department, Wellesley College, Wellesley, Massachusetts, MA, United States; ^3^ Center for Translational Pain Medicine, Department of Anesthesiology, Duke University Medical Center, Durham, NC, United States; ^4^ Department of Anesthesiology of Tri-Service General Hospital & National Defense Medical Center, Taipei City, Taiwan; ^5^ Department of Neurobiology, Duke University Medical Center, Durham, NC, United States; ^6^ Department of Cell Biology, Duke University Medical Center, Durham, NC, United States

**Keywords:** infection, microglia, astrocytes, IFN-α, IFN-β, IFN-γ, spinal cord, nociceptors

## Abstract

Interferons (IFNs) are cytokines that possess antiviral, antiproliferative, and immunomodulatory actions. IFN-α and IFN-β are two major family members of type-I IFNs and are used to treat diseases, including hepatitis and multiple sclerosis. Emerging evidence suggests that type-I IFN receptors (IFNARs) are also expressed by microglia, astrocytes, and neurons in the central and peripheral nervous systems. Apart from canonical transcriptional regulations, IFN-α and IFN-β can rapidly suppress neuronal activity and synaptic transmission *via* non-genomic regulation, leading to potent analgesia. IFN-γ is the only member of the type-II IFN family and induces central sensitization and microglia activation in persistent pain. We discuss how type-I and type-II IFNs regulate pain and infection *via* neuro-immune modulations, with special focus on neuroinflammation and neuro-glial interactions. We also highlight distinct roles of type-I IFNs in the peripheral and central nervous system. Insights into IFN signaling in nociceptors and their distinct actions in physiological vs. pathological and acute vs. chronic conditions will improve our treatments of pain after surgeries, traumas, and infections.

## Introduction

The name of interferons is a reference to their ability to “interfere” with viruses (1). Interferons are a family of cytokines that play important roles in the immune, endocrine, and nervous system, including both the peripheral and central nervous systems (PNS, CNS). Interferons can be divided into three classes: type-I, type-II, and type-III interferons (IFNs). First discovered in 1957, the type-I IFN family consists of IFN-α (IFN-α1-8, 10, 13, 14, 16, 17, and 21, for a total of 13 subtypes), IFN-β, IFN-δ, IFN-ϵ, IFN-κ, IFN-τ and IFN-ω1–3. The type-II IFN family has only one member, IFN-γ (1–4). The pronociceptive and pro-inflammatory role of IFN-γ is well documented. Intrathecal injection of IFN-γ facilitates the spinal nociceptive flexor reflex in rats (5) and induces biting behavior in mice (6). Following nerve injury, IFN-γ contributes to the pathogenesis of murine neuropathic pain through activation of microglia in the spinal cord (7). Type III IFNs are lambda-IFNs, including IFN-λ1, IFN-λ2, and IFN-λ3. Type-I IFNs produce multiple biological and cellular responses, such as suppression of viral infections, inhibition of cell proliferation, promotion of antitumor activities, and modulation of immunity and pain (2, 4, 8, 9).

**Figure 1:**
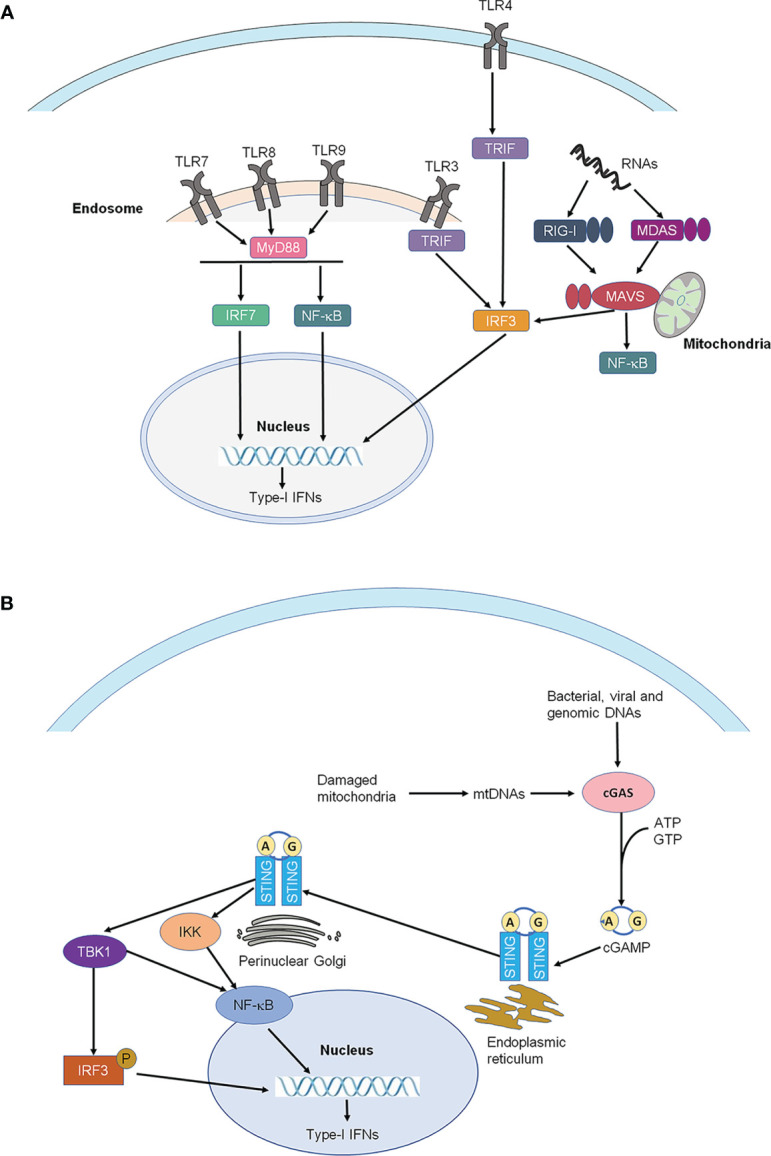
Production of type-I IFNs. **(A)** Production of type-I IFNs by activation of Toll-like receptors (TLRs) and RIG-I-like receptors (RLRs). Type-I IFNs can be induced by the activation of TLR3, TLR4, and TLR7, TLR8, TLR9 *via* distinct intracellular signaling molecules, such as TRIF for TLR3 and TLR4, and MYDd88 for TLR7-9. TLRs are the best-known pattern recognition receptors and are localized either on the cell surface or on intracellular compartments such as endosomes (TLR3, TLR7-9) of immune cells and glial cells. **(B)** Production of type-I IFNs by activation of STING. STING is an endoplasmic reticulum adaptor. Activated by viruses and intracellular DNAs, STING activates TBK1, which subsequently activates the transcription factors NFκB and interferon regulatory factor 3 (IRF3), leading to the production of type-I IFNs. IRF3, interferon regulatory factor 3; IRF7, interferon regulatory factor 7; MAVS, mitochondrial antiviral signaling proteins; MDA5, melanoma differentiation-associated protein 5; MYD88, myeloid differentiation primary response 88; NF-κB, nuclear factor kappa-light-chain-enhancer of activated B cells; RIG-1, retinoic acid-inducible gene; STING, stimulator of interferon genes; TBK1, TANK-binding kinase 1; TRIF, TIR-domain-containing adapter-inducing IFN-β.

It is well-established that the sensing of pathogen-associated molecular patterns (PAMPs) during bacterial or viral infections, or danger-associated molecular patterns (DAMPs) following tissue injury by pattern recognition receptors results in the production of many cytokines and chemokines, including type-I IFNs, leading to innate immunity (10, 11). Toll-like receptors (TLRs) are the best-known pattern recognition receptors and are localized either on the cell surface (e.g. TLR2, TLR5, TLR4) or on intracellular compartments such as endosomes (TLR3, TLR7, TLR8, and TLR9) of immune cells and glial cells (12, 13). TLR3 and TLR7 are activated by double-stranded RNAs and singe-stranded RNAs, respectively, whereas TLR4 is activated by the bacterial product, lipopolysaccharide (LPS) (10). Following the recognition of PAMPs and DAMPs, TLRs will activate their specific signaling pathways to produce type-I IFNs. Notably, type-I IFNs can be induced by the activation of TLR3, TLR4, and TLR7, TLR8, TLR9 *via* distinct intracellular signaling molecules, such as TRIF for TLR3 and TLR4, and Myd88/TRAF3/6 for TLR7-9 (14) (**Figure 1A**).

In addition to TLRs, pattern recognition receptors also include retinoic acid-inducible gene I (RIG)-I-like receptors (RLRs) and stimulator of interferon genes (STING), which sense intracellular RNAs and DNAs, respectively (2 Ivashkiv and Donlin, 2014). There are three receptors in the RLRs family, including RIG-I, melanoma differentiation-associated protein 5 (MDA5), and laboratory of genetics and physiology 2 (LGP2) (14, 15). STING is an endoplasmic reticulum adaptor and regulates intracellular DNA-mediated, type-I interferon-dependent innate immunity (16, 17) (**Figure 1B**). Activated by select viruses and intracellular DNAs, STING activates TANK-binding kinase 1 (TBK1), which subsequently activates the transcription factors NFκB and interferon regulatory factor 3 (IRF3), leading to the production of type-I IFNs and promoting the eradication of pathogens mediated by immune cells and neoplastic cells (18). The plasmacytoid dendritic cells (pDC) are the major type-I IFN-producing cells in humans and mice following virus infection or direct stimulation with DNAs/RNAs (19–21). IFN-α and IFN-β are produced and secreted by different cell types, including natural killer (NK) cells, B cells, T cells, macrophages, fibroblasts, endothelial cells, and osteoblasts. Furthermore, IFN-α and IFN-β can stimulate macrophages and NK cells to elicit anti-viral and anti-tumor responses.

## Type-I Interferon Receptor Signaling in Immune Cells

**Figure 2:**
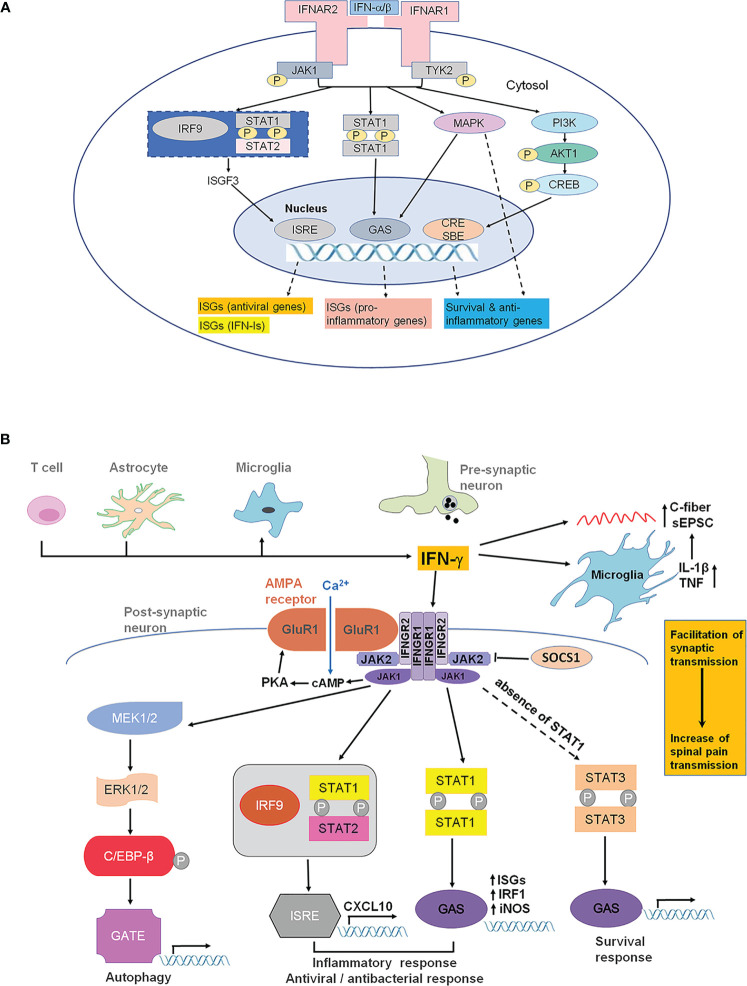
IFNAR and IFNGR signaling. **(A)** IFNAR signaling and type-I IFN-induced gene expression. IFN-α and IFN-β transmit signals through IFNAR1 and IFNAR2 (the main ligand-binding subunit). IFNAR1 and IFNAR2 are distinctly associated with TYK2 and JAK1, respectively. Activation of TYK2 and JAK1 results in activation of STAT1/2, as well as PI3K and MAPK, leading to the transcription of ISGs, including antiviral genes, type-I IFN genes, pro-inflammatory genes, as well as survival and anti-inflammatory genes, through ISRE, GAS, and CRE/SBE. Additionally, TYK2 may be associated with membrane proteins, such as ion channels, causing rapid modulation of cellular function. CRE, cyclic AMP response element; GAS, IFN-γ-activated sites; ISG, interferon-stimulated genes; ISRE, IFN-stimulated response elements; JAK1, Janus kinase 1; MAPK, mitogen-activated protein kinase; PI3K, phosphoinositide 3-kinase; SBE, smad binding elements; STAT, signal transducer and activator of transcription; TYK2, tyrosine kinase 2. **(B)** IFN-γ and IFNGR signaling in the spinal cord for the pathogenesis of pain. IFN-γ can be released by T lymphocytes, astrocytes and microglial cells under pathological pain conditions (e.g., nerve injury). IFN-γ can directly and indirectly alter synaptic activity in spinal dorsal horn neurons and thereby contributes to hyperactivity (central sensitization). IFN-γ facilitates Ca^2+^-permeable AMPA receptors (AMPAR) coupled with IFNGR. These AMPAR lack GluR2 and induce neurotoxicity. IFN-γ can directly activate microglia and increase the release of glial mediators, such as IL-γ and TNF that modulate synaptic strength and facilitated monosynaptic C-fiber-evoked excitatory postsynaptic currents. IFNGR composes of IFNGR1 and IFNGR2 subunits, which are associated with the kinases JAK1 and JAK2. In canonical IFN-γ signaling, phosphorylation of JAK1 and JAK2 results in the phosphorylation of STAT1. A STAT1 homodimer translocates to the nucleus and binds to GAS in the promoters of IFN-γ-regulated genes (IGS) such as NOS-2 and IRF1. In cells that do not express STAT1, STAT3 can be phosphorylated by JAK1/JAK2, resulting in translocation of the STAT3 dimer to the GAS sites.

Both IFN-α and IFN-β bind to a cell surface receptor complex known as the IFN-α receptor (IFNAR) including IFNAR1 and IFNAR2 chains (4, 22, 23). IFNAR is expressed in most cell types including immune cells, neurons and glia in the spinal cord (4, 22, 23). IFNAR signaling requires the Janus activated kinases (JAKs), which consist of 4 family members: JAK1, JAK2, JAK3, and tyrosine kinase 2 (TYK2). IFNAR1 is associated with TYK2, whereas IFNAR2 is associated with JAK1 (**Figure 2A**). Following the ligand-dependent rearrangement and dimerization of receptor subunits, these receptor-associated JAKs auto- phosphorylate for their own activation, leading to subsequent activation of the transcription factor STAT (signal transducer and activator of transcription) (4). The activation of the JAK-STAT signaling pathways appears to mediate the many biological effects of type-I IFNs *via* distinct gene transcription. In contrast, type-II IFN (IFN-γ) binds to IFN-γ receptor (IFNGR), including IFNGR1 and IFNGR2, which are associated with JAK1 and JAK2, respectively (4). Unlike IFN-α and IFN-β, IFN-γ is a prominent pro-inflammatory cytokine and positively regulates neuroinflammation and pathological pain (7). Thus, type-I and type-II IFNs play different roles in inflammation by coupling their target receptors with different intracellular tyrosine kinases.

STATs are activated by IFN receptors and form either homodimers or heterodimers, causing rapid nuclear translocation and gene transcription through binding to appropriate promoter sites on IFN-stimulated genes (**Figure 2A**). Although the JAK-STAT pathway is the most significant pathway for IFN actions, STAT-independent signaling has also been documented (24–26). When binding to specific promoter sites, type-I IFNs can activate the ISG factor 3 (ISGF3) complex, a transcriptional factor that consists of the activated form of STAT1 and STAT2, in addition to IRF9. ISGF3 binds IFN-stimulated response elements (ISREs) to induce the expression of interferon-stimulated genes (ISGs), including antiviral genes and type-I IFNs themselves (**Figure 2A**). Global gene expression profiling studies have demonstrated that type-I IFN administration could induce the expression of hundreds to thousands of genes (27). Besides this canonical signaling pathway, activation of IFNAR1/2 can also result in non-canonical signaling, including the activation of PI3 kinase (PI3K) and mitogen-activated protein kinase (MAPK) pathways (2) (**Figure 2A**).

Type-I IFNs play a crucial role in the functioning of the immune system, mediating and coordinating different gene products to regulate defensive responses against virus infections. Type-I IFNs can induce antiviral gene products to deploy T cells against viruses (3). IFN-β can also activate the suppressor of cytokine signaling (SOCS) protein, which binds to phosphorylated tyrosine residues on JAKs and cytokine receptor chains to inhibit cytokine signaling (25). These cytokines are crucial to the production of both innate and adaptive immune responses. SOCS-1 also inactivates JAK2, decreasing the duration of IFN-γ signaling. This enables the helpful immunological actions of IFN-γ and inhibits the harmful actions of unregulated IFN-γ responses. SOCS-1 can also prevent the expression of the class II major histocompatibility complex (MHC) molecules and CD40, both of which are needed by antigen-presenting cells to activate T cells (28). Administration of type-I IFNs has been shown to be effective in treating several different types of viral infections, including hepatitis C and hepatitis B. Type-I IFNs have also been used to treat autoimmune diseases, such as multiple sclerosis (29), as well as cancers, such as lymphoma and sarcoma (28). Type-I IFNs were proposed as anti-inflammatory mediators by inducing anti-inflammatory cytokines (IL-10) and other immunosuppressive molecules (18), as well as blocking the expression of pro-inflammatory mediators, such as matrix metalloprotease 9 (MMP-9), MHC class II, ICAM-1, VCAM-1, TNF, CXCL8, and IL-12 (30, 31).

However, against acute viral infections, type-I IFNs can cause immunopathology, contributing to various systemic autoimmune diseases, including systemic lupus erythematosus, Aicardi-Goutieres syndrome, rheumatoid arthritis, systemic sclerosis, and Sjogren’s syndrome (28). Furthermore, long-term viral infections can result in high concentrations of type-I IFNs that can inhibit B cell activity or result in the production of immunosuppressive molecules (e.g. IL-10) (18). In the presence of type-I IFNs, activation of TLR4 by LPS can cause IL-10 production (10). Therefore, the pleiotropic effects of type-I IFNs are a double-edged sword, mobilizing immune cells to destroy viruses, bacteria, and other foreign bodies on the one hand, and inducing damaging neuroinflammation on the other hand. The next section will explore both the positive and negative effects of type-I IFNs in the nervous system in more detail. In contrast to type-I IFNs, type-II IFN differentially regulates gene expression and pain (**Figure 2B**).

## IFN-α, IFN-β, and IFN-γ Regulate Pain *via* Neuro-Glial and Neuro-Immune Interactions

Early reports have demonstrated an antinociceptive action of IFN-α in the CNS (**Table 1**). In 1981, Blalock and Smith reported human leukocyte interferon, but not fibroblast interferons, might bind to opioid receptor (32, 43). Furthermore, intracerebral injection of IFN-α caused antinociception and suppression of spontaneous locomotion, which could be reversed by the opioid receptor antagonist naloxone. Unilateral microinjection of IFN-α (4, 8, 16 pmol) into the nucleus submedius, dose-dependently increased the hindpaw withdrawal latency from the noxious heat stimulus in rats, and this effect was specifically mediated by μ-opioid receptor. IFN-α also showed similar pharmacological properties as the opioid peptide β-endorphin (33, 44, 45). However, this early hypothesis of IFN-α binding to opioid receptor remains to be validated.

**Table 1:** Role of IFN-α, IFN-β, and IFN-γ in pain and the underlying mechanisms.

IFNs	Doses	Routes	Species	Conditions	Actions	Mechanisms	References
IFN-α	500 U	Intracerebral	Mouse	Naïve	Antinociception	Opioid receptor dependent	32
	4, 8, 16 pmole	Intracranial ventricle	Rat	Naïve	Antinociception	μ-opioid receptor dependent	33
	100 ng	Intrathecal	Rat	Naïve, CFA	Antinociception	Opioid receptor dependent	9
	100 ng	Intrathecal	Rat	Naïve, CFA	Antinociception	Inhibits EPSC and capsaicin-induced P-ERK	34
	300 U	Intraplantar	Mouse	Naïve	Hyperalgesia	Activation of MAPK and MNK-eIF4e translation	35
	100 U	Intrathecal	Mouse	Naïve	Antinociception	IFNAR-1 mediated actions; inhibition of Nav 1.7 and calcium channel activities	11
IFN-β	100 ng	Intrathecal	Mouse	LPS	Antinociception	IFNAR-1 mediated and TLR-mediated actions	36
	3600 U	Intrathecal	Mouse	Arhtritis	Co-injection with TNF antibody produces long-term pain relief	Induction of IL-10 expression	37
	1000, 5000, 10000 U	Intrathecal	Mouse	Spared nerve injury	Antinociception	Induction of ISG-15 and inhibition of MAPK	38
	300 U	Intraplantar	Mouse	Naïve	Hyperalgesia	Activation of MAPK and MNK-eIF4e translation	35
	100 U	Intrathecal	Mouse	Naïve	Antinociception	IFNAR-mediated actions; inhibition of Nav 1.7 and calcium channel activities	8
IFN-γ	20000 U/mL	Incubation 14d	Rat	Dorsal horn neuron	Increase EPSC	decrease inhibitory neuron GluR 1	39
	1000U	Intrathecal	Rat	Naïve	Hyperalgesia	Activation of IFN-γ receptor in Microglia	7
	1000U	Spinal dorsal nerve	Rat	Naïve	Hyperalgesia	Increase C-fiber response	40
	1000U	Intrathecal	Rat	Naïve	Hyperalgesia	Activation of astrocyte	41
	2000 U/mL	Incubation 4-8h	Rat	Spinal cord slice	Increase C-fiber EPSC	Activation of Microglia	42

In 2012, we reported that intrathecal injection of short-interfering RNAs (siRNAs), at high doses (10 or 20 μg), could produce IFN-α-mediated analgesia, in a rat model of persistent inflammatory pain (9). This finding was very surprising, as the non-targeting siRNAs were used as controls that do not target specific pain genes. This is the first report demonstrating the analgesic effects of non-targeting double-stranded RNAs in the spinal cord, suggesting that caution must be taken when designing siRNAs for target validation in pain research. Notably, short double-stranded RNAs and short hairpin RNAs are able to induce type-I IFN responses in mammalian cells (46). We also found that IFN-α was markedly upregulated in the spinal cord after intrathecal administration of short (<21 bp) double-stranded RNA at a high dose (10 μg) (9). Importantly, the analgesic effect of non-targeting siRNAs can be reversed by intrathecal administration of IFN-α neutralizing antibody, supporting a critical involvement of IFN-α. Furthermore, intrathecal administration of IFN-α (30 and 100 ng) is sufficient to increase paw withdrawal latency in both naïve and inflamed rats in the radiant heat testing, in further support of an antinociceptive action of IFN-α (9).

Increasing evidence suggests that Type-I IFNs regulate pain *via* neuro-immune and neuro-glial interactions. TLR3 can sense double-stranded RNAs to trigger type-I IFN responses *via* TRIF signaling (**Figure 1A**) and regulates pain and itch (13, 46, 47). TLR3 is typically expressed by immune and glial cells (13). Intriguingly, TLR3 is also expressed by nociceptive sensory neurons and modulates nociceptive synaptic transmission in the spinal cord (11, 48, 49). It remains to be tested whether TLR3 is involved in the antinociception induced by the double-stranded RNAs. Von Frey testing revealed that intrathecal IFN-α (100 and 300 U) also increased paw withdrawal threshold in naïve mice (8).

Yaksh and co-workers have also demonstrated an analgesic action of IFN-β: intrathecal injection of IFN-β (100 ng) could relieve tactile allodynia induced by intrathecal TLR2 or TLR4 ligands (36). In a murine model of arthritis, intrathecal IFN-β elicited transient pain relief; however, co-administration of IFN-β and anti-TNF-α antibody produced pain relief for several weeks. This long-term pain relief may result from IFN-β-induced IL-10 expression in the spinal cord of male mice (37). Furthermore, Song and co-workers showed that a single intrathecal IFN-β administration (1000 and 5000 U) could attenuate the nerve injury-induced mechanical allodynia for several days in mice, without affecting open-field activity (38). Mechanistically, this long-lasting effect of IFN-β could be mediated by inhibition of MAPK activation, a key molecular mechanism underlying the pathogenesis of pain (50), and induction of interferon-stimulated gene 15 (ISG-15) after spared nerve injury in mice (38). Intrathecal IFN-β (100 and 300 U) also increased paw withdrawal threshold in naïve mice, in further support of an antinociceptive action of IFN-β (8).

**Figure 3:**
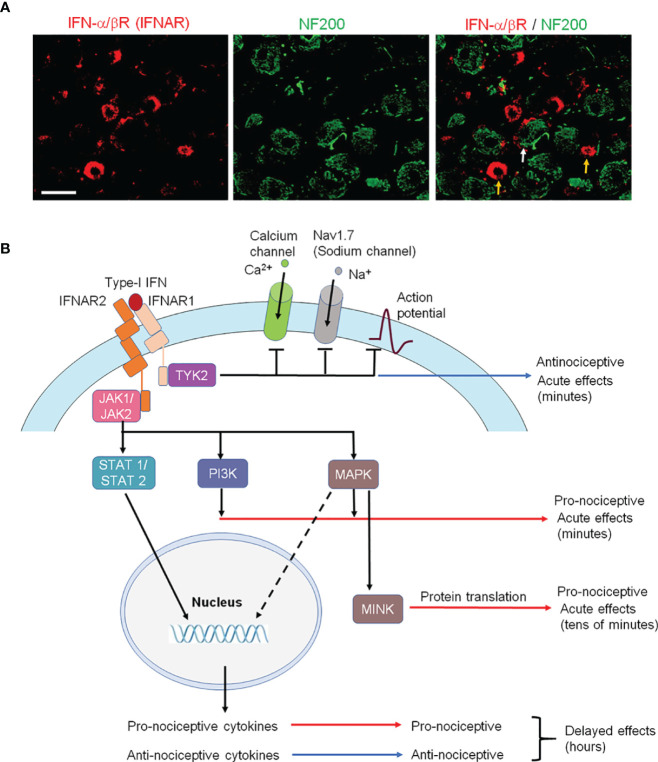
IFNAR signaling in nociceptive sensory neurons and its involvement in pronociceptive and antinociceptive effects. **(A)** Double staining of IFNα/βR (IFNAR) and NF200 in L3-L5 DRG neurons of naïve rats. Note that IFNAR is mainly expressed in NF200-negative nociceptive neurons (orange arrows) and some NF200-postive neurons (white arrow). Scale, 50 μm. Reproduced from Ref 95 (50) with permission. **(B)** Type-I IFN activates IFNAR1, resulting in subsequent TYK2 activation and rapid antinociception *via* inhibition of Na^+^ and Ca^2+^ channels and suppressing of action potential firing in nociceptors at central nervous system. IFNAR-mediated activation of JAK also results in activation of PI3K and MAPK pathways, as well as MINK/eIF4E-mediated translational pathway, leading to nociceptor sensitization at peripheral nervous system. Furthermore, IFNAR-mediated STAT activation causes delayed effects *via* induction of pro-inflammatory and anti-inflammatory cytokines, leading to pro-nociceptive and anti-nociceptive actions in the late-phase at central nervous system.

*In situ* hybridization and single-cell RNAseq analyses revealed broad expression of *Ifnar1* mRNA in various types of primary sensory neurons of dorsal root ganglion (DRG) of mice (8, 35, 49, 51). Furthermore, immunohistochemistry revealed the expression of IFN-α/β receptor (IFNAR) has limited expression in large fiber which expressed NF200, a cellular marker for large-myelinated neurons (**Figure 3A**), but expresses extensively in mouse DRG C-fiber nociceptive neurons that co-express the neuropeptide CGRP (34). Notably, LPS-induced tactile allodynia is prolonged in mice lacking *Ifnar1* and this LPS-induced mechanical pain did not show any sign of recovery at 21 days of LPS in *Ifnar1* null mice (36). Strikingly, selective deletion of *Ifnar1* in Nav1.8-expressing nociceptors led to pain hypersensitivity and increased excitability of nociceptor neurons of naïve mice, suggesting an essential role of type-I IFN signaling in the control of nociception under the homeostasis condition (8). Conversely, incubation of dissociated wild-type DRG neurons with type-I IFN (30 U/ml) suppressed nociceptor excitability by inhibition of Nav1.7-mediated sodium currents and calcium currents (**Figure 3B**) (8).

**Figure 4:**
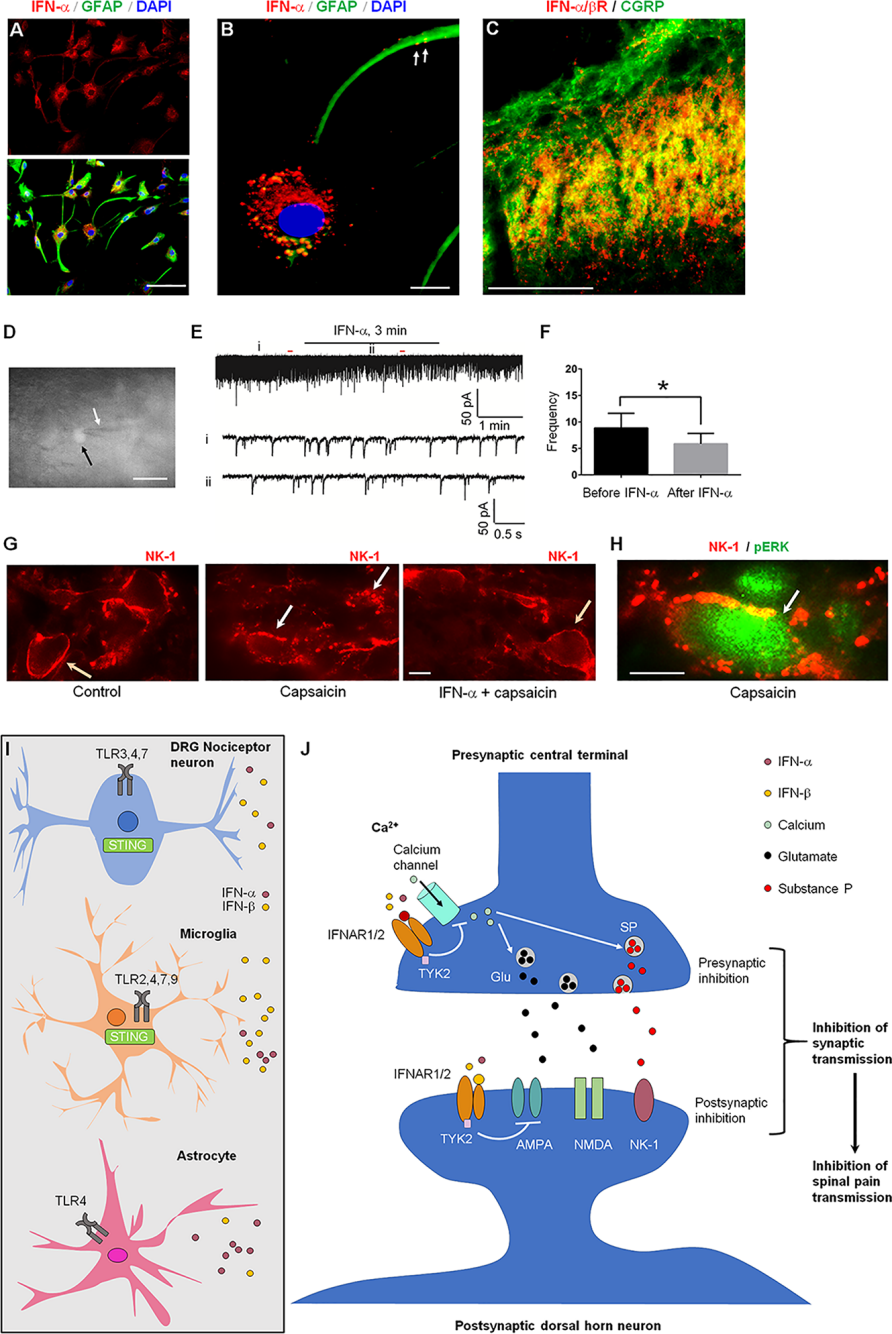
IFNAR signaling in the spinal cord for the inhibition of nociception. **(A)** IFN-α expression in cultured astrocytes. **(B)** Triple staining of IFN-α, GFAP, and nuclei marker DAPI in cultured astrocytes prepared from cerebral cortexes of neonatal Sprague-Dawley rats. Scales, 50 μm in A and 10 μm in B. Arrows indicate IFN-α-labeled vesicles in remote astrocyte processes. **(C)** Double staining of IFNAR (IFN-α/βR) and CGRP in the superficial dorsal horn. Scale, 100 μm. **(D–F)** IFN-α inhibits synaptic transmission in spinal cord slices. **(D)** The recorded neuron (somatostatin-positive, shown by white arrow) with an electrode (black arrow). **(E)** Patch clamp recording reveals an inhibition of spontaneous excitatory postsynaptic currents (sEPSCs) in lamina II neurons by IFN-α (25 ng/ml, 2 min). i and ii indicate traces before and after the treatment. **(F)** Frequency (Hz) of sEPSCs. 7 out of 9 recorded neurons respond to IFN-α. *P<0.05, n = 7 neurons. **(G, H)** IFN-α suppresses spinal nociceptive transmission. **(G)** Intraplantar capsaicin-evoked NK-1 internalization in superficial dorsal horn neurons is suppressed by intrathecal IFN-α (100 ng). The slices were stimulated with capsaicin (3 mM, 5 min) or pretreated with IFN-α 10 min before capsaicin stimulation. Orange and white arrows indicate surface-expressed and internalized NK-1 receptors, respectively. **(H)** Double staining showing co-localization of NK-1 (with internalization) and ERK phosphorylation (pERK) in a lamina I neuron (arrow) following capsaicin stimulation. Scales, 10 μm. A-H are reproduced from (50) with permission. **(I, J)** Type-I IFN and IFNAR signaling mediates neuro-glial interactions in the spinal cord for pain modulation. **(I)** IFN-α and IFN-β are produced by astrocytes, microglia, and nociceptive neurons following stimulation of TLRs and STING. **(J)** Activation of IFNAR inhibits nociception *via* both pre-synaptic and post-synaptic mechanisms. At the presynaptic sites, IFNAR activation inhibits calcium channels and neurotransmitter release. At the post-synaptic sites, IFNAR activation inhibits the activity of the glutamate AMPA receptors.

In the rat spinal cord, IFN-α is mainly expressed by astrocytes that express glial fibrillary acidic protein (GFAP, **Figure 4A**) (9, 34). IFN-α is also found in vesicle-like structures of astrocytic processes in primary cultures (**Figure 4B**), suggesting a vesicle-mediated release from astrocytes. Interestingly, IFN-α/β receptor (IFNAR) was found to be expressed in spinal cord axonal terminals co-expressing CGRP (**Figure 4C**). The result indicated a neuronal expression of IFNAR in presynaptic terminals (34), although we could not exclude IFNAR expression in other cell types. A direct modulation of synaptic transmission in the spinal cord nociceptive circuit by IFN-α has been demonstrated by *ex vivo* electrophysiology in spinal cord slices (**Figures 4D–F**) (52). Perfusion of spinal cord slices with IFN-α (50 U/ml, 3 min) resulted in a rapid suppression of excitatory synaptic transmission by reducing the frequency of spontaneous excitatory postsynaptic currents (sEPSCs) in outer lamina II (IIo) excitatory interneurons expressing somatostatin (53). IFN-α also blocked the capsaicin-induced central sensitization (52), as revealed by 1) internalization of neurokinin-1 (NK-1) receptor and 2) phosphorylation of extracellular signal-regulated kinase (ERK) in superficial dorsal horn neurons (**Figures 4G, H**). When naive rats or mice were intrathecally injected with a neutralizing antibody to remove endogenous IFN-α or IFN-β, it resulted in hyperalgesia (8, 9). Activation of TLR3 by Poly (I:C) upregulates IFN-β in primary cultures of microglia and astrocytes (36). Additionally, *Ifnar1* null mice exhibited increased EPSCs in the spinal cord pain circuit, compared with wild-type littermates (8). Thus, the respective expression of IFN-α and IFNAR in glial cells and neurons has provided a cellular basis for spinal cord neuro-glial interactions in regulating type-I IFN-mediated nociception (**Figures 4I, J** **)** (52).

Recently, Donnelly et al. (8) reported STING-mediated antinociception in mice and nonhuman primates, which is governed by type-I IFNs (8). Strikingly, type-I IFN can rapidly suppress the excitability of mouse, monkey, and human nociceptors (8). Intrathecal injections of STING agonists produce sustained anti-nociception (24-48 h) in naïve mice, as well as mice with neuropathic pain and cancer pain, without showing signs of motor impairment. This antinociception by STING agonists is associated with increased expression of IFN-α and IFN-β in DRG and spinal cord tissues and abolished in *Ifnar1* knockout mice. Notably, *Sting* knockout mice, as well as conditional knockout mice with selective deletion of *Sting* in nociceptor neurons, can recapitulate the phenotypes of *Ifnar1* knockout mice, showing drastically increased pain sensitivity and neuronal hyperexcitability (8). *Sting* mRNA is expressed by both nociceptor neurons of DRG and microglia of spinal cord, suggesting that type-I IFNs can be produced by both neurons and glia (8) (**Figure 4I, J** **)**.

There were also recent reports showing pronociceptive actions of type-I IFNs (35, 54, 55) (**Table 1**). Barragán-Iglesias, P. et al. found that intraplantar administration of either IFN-α or IFN-β (300 U in 25 μl), but not vehicle (saline), produced a persistent mechanical hypersensitivity to von Frey filament stimulation in mice, lasting for days. Furthermore, this persistent hyperalgesia depends on type-I IFN-induced MAP kinase activation and MNK/eIF4E-mediated translation in DRG neurons (35). Moreover, exposure of mouse DRG neurons to IFN-α (300 U/ml) increased nociceptor excitability (35). It is unclear if these pronociceptive actions of type-I IFN are mediated by IFNARs. Interestingly, mechanical hypersensitivity induced by high-dose intraplantar IFN-α can be blocked by intrathecal IFN-α (8), suggesting distinct actions of peripheral vs. central IFN-α. IFNAR1 and IFNAR2 are also widely expressed by vagal sensory neurons, including 70% of the TRPV1-positive neurons (presumably nociceptors). Calcium imaging revealed an acute activation of bronchopulmonary vagal nociceptors by IFN-α and IFN-β at high concentrations (1000 and 10,000 U/ml) (55). Another study showed that formalin-evoked nociceptive behavior is potentiated by systemic IFN-α administration for 8 days at very high doses (8000 IU/gram body weight/day) (54). It is important to note that peripheral IFN-β normally does not directly penetrate BBB to the CNS (56). Thus, the pronociceptive effects induced by intraplantar or systemic administration of type-I IFN is a result of peripheral and/or local actions. Hence, it is conceivable that the discrepancy of antinociceptive vs. pronociceptive effects of type-I IFNs may result from 1) location difference (peripheral vs. central) or 2) concentration difference (low vs. high) of type-I IFNs. Interestingly, TYK2 was implicated in the antinociceptive actions of type-I IFNs, and intrathecal TYK2 inhibitor blocked the STING agonist-induced analgesia and further induced hyperalgesia in naïve mice (8). It is also likely that distinct activation of downstream signaling pathways (e.g., TYK2 vs. PI3K/MAPK pathway) may lead to different regulations of neuronal excitability, as the activation of the PI3K/MAPK pathway is known to cause nociceptor sensitization (57, 58) (**Figure 3B**).

It is also noteworthy that high doses of type-I IFN treatment may generate autoantibodies, as anti-IFN-β antibodies did appear in a portion of MS patients (3-45%), especially in patients receiving subcutaneous formulation (59). IgG autoantibodies and the corresponding antigens can form the IgG-IC complex that can directly bind to the neuronal Fc-gamma receptor (FcγR) to activate nociceptors and promote pain (60). Neuromyelitis optica is an antibody-mediated autoimmune inflammatory disease of the CNS, caused by the loss of aquaporin-4 and GFAP that are mainly expressed by astrocytes. Type-I IFN contributes to the disease progression, as loss of aquaporin-4 and GFAP following intracerebral injection of IgG from a neuromyelitis optica patient was alleviated in mice lacking IFNAR (61). Patients with neuromyelitis optica also suffer from severe pain due to dysregulation of astrocytes and generation of aquaporin-4 autoantibodies (52, 62).

As the only Type-II IFN, IFN-γ was firstly reported to be able to inhibit viral replication (63). IFN-γ is released by subsets of NK cells, glial cells and activated T lymphocytes during infection and inflammation in the nervous system (64). IFN-γ not only has antiviral activity but also is involved in the polarization of the immune response and the regulation of macrophage plasticity by producing “M1” phenotype and release of pro-inflammatory cytokines such as IL-1β, IL-12, IL-23, and TNF-α (65). In general, IFN-γ signaling is short-lived to elicit functional recovery of homeostasis, including tissue repair and reestablishment of tissue physiology. Different from type 1 IFN binding to IFNAR1 and IFNAR2, IFN-γ signaling is mediated by a distinct receptor composed of the two subunits IFNGR1 and IFN-rR2 in a four-chain assembly (66) (**Figure 2B**), whose signal transduction cascade involves the activation of JAK1, JAK2 and the phosphorylation of STAT1(pSTAT1), STAT3, STAT5 and the indirect activation of the NF-kB module (67). pSTAT1 binds with high affinity to DNA sequences termed the γ-interferon-activated site (GAS) to initiate transcription of interferon-stimulated genes (ISG), iNOS and IRF1. Phosphorylation of C/EBPβ induced by IFN-stimulated proteolytic processing of ERK1 and ERK2 is necessary to control autophagy of infectious agents. Prolonged activation of IFN-γ resulted in the formation of the heterotrimeric transcription factor complex of STAT1 and IFN-regulatory factor 9 (Irf9), leading to altered expression of CXCL10. Non-canonical pathways could be activated in the absence of STAT1. For example, IFN-γ can activate STAT3 and subsequently activate GAS-regulated genes through JAK-STAT-dependent pathway. STAT-independent IFN-γ signaling can also occur *via* activation of MAPK signaling pathways. For example, IFN-γ can activate ERK1/2 to trigger binding of CCAAT/enhancer-binding protein-β (C/EBPβ) to a novel IFN-γ response element (GATE)(**Figure 2B**) In the CNS, IFNGR1/2 is expressed on neurons and glial cells in the spinal cord dorsal horn (6, 7, 68–70). IFN-γ is secreted by glial cells, neurons and infiltrating monocytes and is involved in promoting neuroinflammation but also neuroprotective processes such as neurogenesis and brain repair (64). Upregulation of IFN-γ was observed in the lumbar spinal dorsal horn after nerve injury (71–73). Intrathecal application of IFN-γ could induce persistent pain behavior in rodents (6, 7, 41, 72, 74), increase spinal nociceptive reflexes (75), evoked immediate biting behavior in rats (6). IFN-γ increased phospho-STAT1 levels in the spinal dorsal horn within minutes (7). Loss of IFNGR results in attenuation of tactile allodynia in mice after nerve injury (7, 71).

IFNGR has been reported to be located predominantly in postsynaptic sites on dendrites in lamina I and II and in axon terminals in the lateral spinal nucleus (68). Treatment of cultured dorsal horn neurons with IFN-γ resulted in enhanced spontaneous postsynaptic excitatory current (sEPSC), as well as reduction of AMPAR clustering on inhibitory interneurons, leading to disinhibition (39). Interestingly, disinhibition can also be induced by the pro-inflammatory cytokine IL-1β (76). Furthermore, prolonged but not acute treatment with IFN-γ facilitated monosynaptic C-fiber-evoked excitatory postsynaptic currents and this effect could be blocked by the application of minocycline, an inhibitor of microglial activation, suggesting an essential involvement of microglia (42) (**Figure 2B**). Stimulation of IFNGR in spinal microglia under normal conditions is sufficient to activate microglia and induce mechanical allodynia. By contrast, loss of IFNGR impaired nerve injury-induced microglia activation (7). However, another study found activation of spinal microglia after nerve injury was not completely eliminated in IFNGR-deficient mice, suggesting the involvement of IFNGR-independent mechanisms for the activation of microglia (77). Interestingly, local upregulation of IFN-γ in nucleus pulposus following disc herniation was associated with acute lumbar redicular pain (40). In this study, a significant increase in C-fiber response following application of NP and administration of IFN-γ on the dorsal nerve roots was observed. Interestingly, the single nucleotide polymorphisms (SNPs) rs2069705 in the promoter and rs2069718 in intron 3 may cause excessive expression of IFN-γ in systemic lupus erythematosus (SLE) patients (78, 79). A significant increase in Oswestry Disability Index (ODI) score, assessing physical function related to low back pain, was observed in patients with the IFN-γ rs2069705 and rs2069718 genotype (40). The potential mechanisms of disc herniation induced-neuropathic pain are central sensitization and *via* IFNG-GluR1 complex-GluR1 complex activated by IFN-γ (39, 80) (**Figure 2B**, **Table 1**).

## Roles of Type-I IFNs in Infectious Diseases and COVID-19

Type-I IFNs play a major role in controlling encephalitis. During sterile neuroinflammation, microglia take on a pathological role; however, in viral encephalitis, their role remains unclear. Viruses can enter the CNS through the olfactory bulb (81). After intranasal vesicular stomatitis virus instillation, one study found increasing accumulation of microglia and monocytes in the olfactory bulb. Microglia depletion during encephalitis enhances virus spread, leading to increased lethality (81). Another study reported that type-I IFN signaling in astrocytes confers protection against viral encephalomyelitis and mediates IFN-γ-dependent responses (82). Furthermore, the type-I IFN receptor signaling on neurons has an important role in the activation of myeloid cells. In the infected CNS, communication between neurons, astrocytes, and microglia is essential to prevent encephalitis from becoming lethal (81). There is increased secretion of IFN-α and IFN-β in viral encephalitis (83) and secretion of IFN-α by microglia and astrocytes in human immunodeficiency virus-1 (HIV-1)-associated encephalitis was also reported (84). Aicardi-Goutières syndrome (AGS) is a genetic inflammatory disorder that affects the brain (encephalopathy), characterized by an increase in IFN-α levels in the brain. Interestingly, astrocytes were found to be the major source of IFN-α in AGS (85). Additionally, neurons differentiated either from the human NTera-2 cell line or isolated from mice infected *in vivo* with Theiler’s virus or La Crosse virus, can produce IFN-β (86). Human NTera-2 cell line contains pure neurons that progressively develop an extensive network of neurites and did not give rise to any glial cells, as tested by RT-PCR. Notably, neurons can produce IFN-α and IFN-β, in addition to macrophages/microglial cells and ependymal cells in mice infected by Theiler’s virus or La Crosse virus (83). However, fewer than 3% of infected neurons expressed type-I IFNs, suggesting limited production of type-I IFNs by neurons (83). It is noteworthy that IFN receptors are ubiquitously expressed in various cell types, such as macrophages, monocytes, T lymphocytes, glia, and neurons (1, 87). Following viral infections, a compliment-microglial axis results in synapse loss and memory impairment (88, 89), likewise the IL-1 and IL-1R axis inhibits adult neurogenesis and causes memory dysfunction in mice (90). Double stranded RNA is generated during viral replication and has been shown to drive anti-viral innate immune responses, sickness behavior and cognitive dysfunction, which is dependent on IFNAR1 expression and age (91). Interestingly, increased IFN-α levels were found in the CSF of HIV patients, which may contribute to HIV-induced dementia (84).

Type-I IFNs are also being examined for their role in the ongoing coronavirus disease 2019 (COVID-19) pandemic, resulting from the serve acute respiratory syndrome coronavirus 2 (SARS-CoV-2). Type-I IFNs appear to be protective in the early stages of COVID-19. Park et al. proposes that human coronaviruses can produce viral proteins (e.g. a highly basic nucleocapsid protein (92) that inhibit IFNAR signaling, resulting in a diminished IFN response to inflammation, contributing to the pathogenesis of the disease. Type-I IFN autoantibodies may also contribute to severe COVID-19 (93, 94). Conversely, patients with severe COVID-19 showed elevated levels of IFN-α that correlated with disease severity and persisted even after disease resolution (95). IFN-β treatment has been proposed to treat patients in early stages of COVID-19, especially if applied at the site of infection, while immunomodulatory drugs may help reduce inflammation in patients in late stages of COVID-19 (96). Furthermore, type-I IFN response could exacerbate inflammation in patients with severe COVID-19, pointing to the coexistence of type-I IFN and TNF/IL-1β-driven inflammation in patients with severe COVID-19 (97). Delirium is a serious disturbance in mental abilities that is associated with confused thinking. It is noteworthy that delirium developed in 30% COVID-19 patients and was associated with increased mortality (98, 99). Cancer patients with neurologic sequelae of COVID-19 express leptomeningeal inflammatory cytokines in the absence of viral neuroinvasion, with elevated levels of IFN-γ in plasma and IFN-β in CSF (100).

Viral infections can either produce pain or inhibit pain in a context-dependent manner. Accumulating evidence suggests that nociceptors express pattern recognition receptors such as TLR3, TLR4, TLR5, and TLR7/TLR8 that can directly sense the presence of viruses, and viral infections are frequently associated with acute pain (11, 101, 102). Several types of viruses, such as herpes simplex virus (HSV) and varicella zoster virus can effectively infect sensory neurons, and in a latent phase, these viruses could be reactivated and produce intense and persistent pain through inducing skin inflammation and generating pro-inflammatory mediators (11, 103). It is also important to point out that infections can both produce pain and inhibit pain (11). HSV has been used as a gene therapy vector to deliver anti-inflammatory genes encoding IL-10 and IL-4 for sustained pain relief (104, 105). Intraplantar injection of HSV-1 increased mechanical sensory thresholds in rats (106). Furthermore, in early studies infection of rodent DRG neurons with HSV-1 or HSV-2 was shown to suppress sensory neuron excitability and sodium currents (107, 108). Intriguingly, patients not only feel pain but also experience paresthesia (numbness, tingling) after reactivation of HSV and subsequent ulceration. Thus, infections may inhibit or potentiate pain depending on the stage of the infection and distinct actions of viruses on immune cells and neurons (11, 102). Notably, many COVID survivors will also end up with chronic pain (109). The specific role of Type-I IFNs in pain sensitivity changes associated with viral infections remain to be investigated.

## Concluding Remarks, Clinical Relevance, and Future Directions

Type-I IFNs, including IFN-α and IFN-β, are cytokines produced by almost every type of cell in the CNS and PNS. Type-I IFNs are essential for protecting the host against infection and have been used to treat different types of viral infections, autoimmune diseases, and cancers (28, 29). For example, recombinant IFN-β is currently used as treatment for relapsing-remitting multiple sclerosis (110, 111) and recombinant IFN-α is used to treat hepatitis (112). Systemic IFN-α therapy decreased bone metastases and increased metastasis-free survival in a murine model of breast cancer metastasis (113). Although type-I IFNs are involved in CNS inflammation and neurological disease, they can produce either protective or detrimental effects depending on the disease conditions. Inappropriate or chronic production of type-I IFNs by cells in the CNS can induce a number of diseases, including autoimmune diseases and chronic and congenital infections (114, 115), generally called “cerebral interferonopathies” (116). Prolonged exposure to type-I IFNs was reported to induce sickness behavior, including depression, anxiety, cognitive impairment, and even delirium in both mice and humans (56, 117).

IFNs are the best-known cytokines that are induced by virus infection to fight against infection, and a recent study shows that type-I IFN production is controlled by STING in the pain pathway and the STING/type-I IFN axis is present in the normal DRGs and controls physiological pain under the homeostasis condition. The role of type-I IFNs in pain is still controversial, as both pronociceptive actions (35, 55) and antinociceptive actions (8, 9, 33, 34, 36–38) of IFN-α and IFN-β have been reported, depending on the locations and doses (**Table 1**). This discrepancy may also depend on conditions (physiological vs. pathological) and phases (acute vs. chronic).

In summary, IFNs play important physiological roles in the nervous system, regulating various brain functions, including nociception. In the spinal cord, IFN-γ promotes pain while IFN-α and IFN-β inhibit nociception. IFN-α and IFN-β have been used to treat infectious disease, cancer, and MS. However, chronic production of IFNs contributes to chronic neuroinflammation, which is associated with aging, cognitive decline, and neurological diseases. Accumulating evidence suggests that IFN signaling is also critical for neuro-glial interactions in physiological and pathological conditions. New insights into neuro-glial modulations by IFNs may lead to new therapeutics for the control of pain and neurological diseases.

## Author Contributions

P-HT contributed to the second part (pain modulation), the last part (clinical relevance), **Figures 2**–**4**, and **Table 1** of this review. He also obtained the permission of reproducing **Figure 3A** and **Figure 4**. JJ contributed to the first part (introduction) and second part (IFNAR signaling) and prepared **Figures 1** and **2A** of the review. C-CY edited the manuscript. R-RJ edited all the sections and figures/tables. All authors contributed to the article and approved the submitted version.

## Funding

The manuscript was partly supported by grants from the Taiwan Ministry of Science and Technology (MOST 108-2314-B-384 -008 -MY3), Chi Mei Hospital Grants (CMFHR 10902, CMNDMC11012).

## Conflict of Interest

R-RJ is a consultant of Boston Scientific and received a research grant from the company. This activity is not related to this study.

The remaining authors declare that the research was conducted in the absence of any commercial or financial relationships that could be construed as a potential conflict of interest.

## Publisher’s Note

All claims expressed in this article are solely those of the authors and do not necessarily represent those of their affiliated organizations, or those of the publisher, the editors and the reviewers. Any product that may be evaluated in this article, or claim that may be made by its manufacturer, is not guaranteed or endorsed by the publisher.
